# N6-methyladenosine within transmissible gastroenteritis virus genomic RNA inhibits its replication via efficient recognition by RNA sensor RIG-I

**DOI:** 10.1128/jvi.01373-25

**Published:** 2025-12-31

**Authors:** Jianing Chen, Shengyu Lin, Qianzi Liu, Mengling Gao, Zemei Wang, Jiao Tang, Yaru Cui, Chen Tan, Guangliang Liu

**Affiliations:** 1 State Key Laboratory of Animal Disease Control and Prevention, College of Veterinary Medicine, Lanzhou University, Lanzhou Veterinary Research Institute, Chinese Academy of Agricultural Sciences, Lanzhou, China; 2 College of Animal Sciences, Fujian Agriculture and Forestry University 12449, Fuzhou, Fujian, China; 3 College of Veterinary Medicine, Gansu Agricultural University 739715, Lanzhou, Gansu, China; 4 College of Veterinary Medicine, Shanxi Agricultural University, Jinzhong, Shanxi, China; University of Kentucky College of Medicine, Lexington, Kentucky, USA

**Keywords:** N6-methyladenosine, transmissible gastroenteritis virus, coronavirus, RIG-I, post-transcriptional modification

## Abstract

N6-methyladenosine (m^6^A) is the most abundant internal modification in eukaryotic RNA and plays diverse roles in RNA metabolism. Increasing evidence indicates that m^6^A is also present in viral RNAs, where it exerts virus-specific effects. While several studies have shown that m^6^A can facilitate viral replication, its antiviral mechanisms remain less understood. In this study, we used transmissible gastroenteritis virus (TGEV) as a model to investigate the inhibitory role of m^6^A in viral infection. We demonstrated that m^6^A modification is present in the TGEV genome and suppresses viral replication. The m^6^A reader proteins bind to viral RNA and reduce the stability of m^6^A-modified transcripts. Notably, TGEV infection increased global m^6^A levels in host RNA, particularly in interferon (IFN)-associated genes. Inhibition of m^6^A methylation significantly diminished IFN gene expression. Furthermore, compared to other viruses, TGEV genomic RNA displayed an abnormally higher m^6^A ratio, which can be distinguished by RIG-I to promote an immune response. Collectively, our findings reveal that high m^6^A modification enhances RIG-I-mediated sensing of TGEV RNA, leading to the activation of IFN responses and inhibition of viral replication. This study provides new insights into the complex regulatory functions of m^6^A during viral infection and host antiviral defense.

**IMPORTANCE:**

N6-methyladenosine (m^6^A) is one of the most prevalent RNA modifications in viral genomes, but its functional impact varies widely across viruses. While m^6^A often promotes viral replication, it can exert inhibitory effects in certain viruses, particularly within the *Flaviviridae* and *Coronaviridae* families. Despite growing evidence of this antiviral role, the underlying mechanisms remain largely unclear. Here, we used transmissible gastroenteritis virus (TGEV), a swine coronavirus, as a model to explore the inhibitory function of m^6^A. We show that the TGEV genome harbors a relatively high density of m^6^A modification compared to other viruses and host mRNA, which are efficiently detected by the host pattern recognition receptor RIG-I. This interaction enhances innate immune activation and restricts viral replication. Our findings uncover the mechanism by which abnormal m^6^A modification can be sensed to activate antiviral immunity and provide deeper insight into the multifaceted role of m^6^A in host–virus interactions.

## INTRODUCTION

N6-methyladenosine (m^6^A) is the most abundant internal modification found in eukaryotic mRNA, playing essential roles in RNA metabolism, including splicing, export, translation, and stability (1, 2). This dynamic modification is regulated by a set of proteins: “writers” (methyltransferases), “erasers” (demethylases), and “readers” (m^6^A-binding proteins). The methyltransferase complex primarily consists of methyltransferase-like 3 (METTL3) and METTL14, with additional regulators, such as WTAP, KIAA1429, RBM15, and ZC3H13 (3–7). Demethylation is mediated by enzymes such as FTO, ALKBH3, and ALKBH5 (8–10). The functional consequences of m^6^A depend largely on its recognition by reader proteins, particularly the YTH domain family members: YTHDF1, YTHDF2, and YTHDF3. These proteins influence mRNA fate by modulating translation efficiency and RNA decay (11–13).

Although m^6^A was first identified on viral RNA transcripts as early as the 1970s (14–18), its functional significance during viral infection was not fully appreciated until the advent of transcriptome-wide m^6^A mapping technologies in 2016 (19–21). These studies revealed the widespread presence of m^6^A across the RNA genomes of various viruses and highlighted its functional relevance in infection. For many RNA viruses—including HIV-1, enterovirus 71 (EV71), influenza virus, respiratory syncytial virus (RSV), and vesicular stomatitis virus—m^6^A modification promotes replication (22–26). Several DNA viruses, such as hepatitis B virus, herpes simplex virus, Kaposi’s sarcoma-associated herpesvirus (KSHV), and adenoviruses, also benefit from m^6^A modification (27–30). However, in some contexts, such as with KSHV, the effects of m^6^A appear to be cell type-dependent (31).

To date, only members of the *Flaviviridae* and *Coronaviridae* families have been consistently reported to be negatively regulated by m^6^A (32–35). The mechanisms underlying this antiviral effect remain incompletely understood.

Transmissible gastroenteritis is a highly contagious enteric disease in swine, characterized by high mortality in neonatal piglets (36). The causative agent, transmissible gastroenteritis virus (TGEV), is a positive-sense, single-stranded RNA virus approximately 28.5 kb in length, belonging to the *Alphacoronavirus* genus of the *Coronaviridae* family. The TGEV genome features a typical coronavirus organization: 5′UTR–ORF1a/1b–S–ORF3a/3b–E–M–N–NS7–3′UTR. Although TGEV is currently rare in Europe and sporadic in Asia (37, 38), it has contributed genetic material to other emerging coronaviruses through recombination, including porcine epidemic diarrhea virus (PEDV), canine coronavirus II, and CCoV-HuPn-2018 (39–41). Thus, TGEV continues to be relevant as a model virus and potential source of zoonotic genetic elements.

In this study, we explored the role of m^6^A modification in the replication and immune recognition of TGEV. We found that TGEV genomic RNA is extensively methylated at m^6^A sites, which reduces RNA stability through YTH domain-containing reader proteins and activates a potent interferon response via RIG-I. These findings reveal a dual mechanism by which m^6^A modification suppresses TGEV replication and enhances our understanding of m^6^A-mediated regulation in host–virus interactions.

## MATERIALS AND METHODS

### Cell culture and virus infection

PK-15 cells were cultured in Dulbecco’s Modified Eagle Medium (DMEM; Sigma, Germany) supplemented with 5% calf bovine serum (CBS; Sigma, Germany) and 100 U/mL penicillin (Sigma, Germany) at 37°C in a humidified incubator with 5% CO₂. The TGEV-H strain, a cell-adapted variant, and the field-isolated TGEV-QY18 strain were obtained and maintained in our laboratory.

### Antibodies

The following primary antibodies were obtained from commercial sources: mouse anti-Myc mAb (ABT2065, Abbkine), anti-GAPDH mAb (ABL1020, Abbkine), anti-β-actin mAb (ABL1010, Abbkine), rabbit anti-m^6^A pAb (202 003-50, Synaptic Systems), rabbit anti-FTO mAb (ab126605, Abcam), rabbit anti-METTL3 mAb (ab195352, Abcam), rabbit anti-METTL14 mAb (ab309096, Abcam), rabbit anti-YTHDF1 mAb (ab220162, Abcam), rabbit anti-YTHDF2 mAb (ab220163, Abcam), rabbit anti-YTHDF3 mAb (ab220161, Abcam), rabbit anti-RIG-I mAb (3743, CST), rabbit anti-IRF3 mAb (4302, CST), rabbit anti-*p*IRF3 mAb (29047, CST), rabbit anti-MDA5 mAb (5321, CST).

### Virus titration

For virus titration, PK-15 cells were seeded into 96-well plates. Supernatants from infected cultures were collected, serially 10-fold diluted, and inoculated into quadruplicate wells. After 48 h of incubation, cells were fixed with 4% paraformaldehyde for 30 min, permeabilized with 0.5% Triton X-100 for 15 min, and blocked with 5% skim milk for 1 h. The cells were then incubated with a home-made mouse monoclonal antibody against the TGEV nucleocapsid (1:5,000 dilution) for 1 h, followed by Alexa Fluor 488-conjugated goat anti-mouse IgG secondary antibody (1:1,000 dilution; Abbkine, China). Fluorescence was visualized under a TE2000U fluorescence microscope (Nikon, Japan) equipped with a digital documentation system. Viral titers were calculated using the Reed–Muench method.

### Plaque assay

PK-15 cells were seeded in 12-well plates and pre-treated with various concentrations of 3-deazaadenosine (3-DAA) (Macklin, China) for 1 h before infection. Upon reaching confluence, cells were infected with TGEV at a multiplicity of infection (MOI) of 0.01. After 1 h of adsorption, cells were overlaid with 0.8% low-melting-point agarose (Sigma, Germany) in DMEM supplemented with 5% CBS and 3-DAA. After 24 h of incubation at 37°C, plaques were visualized by staining with 1% crystal violet in ethanol.

### RNA interference

For the knockdown of genes involved in the process of methylation, siRNAs against different genes were synthesized (RiboBio, China) and transfected into PK-15 cells using X-tremeGENE siRNA Transfection reagent (Roche, Switzerland) at a ﬁnal concentration of 100 nM. The cells were harvested at 48 hours post transfection ( hpt) for western blot analysis. The target sequences of the siRNAs are listed in Table 1.

**TABLE 1:** The target sequences of siRNAs

Gene name	Species	Target sequence
FTO	*Sus scrofa*	GCACCTACAAGTACCTGAA
METTL 3	*Sus scrofa*	CTGAACCAACAATCTACTA
METTL 14	*Sus scrofa*	AGAGACAGATGAAGACAAA
YTHDF1	*Sus scrofa*	CTCCGCCCATAAAGCATAA
YTHDF2	*Sus scrofa*	CAAGGAAACAAAGTGCAAA
YTHDF3	*Sus scrofa*	GGGAGAGAAATAGAAACAA

### MeRIP and M^6^A-seq

Methylated RNA immunoprecipitation (MeRIP) and m^6^A-seq were performed as described previously (34). Briefly, the total RNA was extracted from cells and depleted of rRNA using the RiboMinus Eukaryote System v2 (Thermo, USA). The RNA was fragmented and incubated with anti-m^6^A polyclonal antibody (Synaptic Systems, Germany) at 4°C for 2 h. Immunoprecipitated and input RNAs were used for library construction with the TruSeq RNA Library Prep Kit (Illumina, USA), followed by sequencing on an Illumina HiSeq 2000 platform (Shanghai Jiayin Biotechnology Ltd.). The raw data are publicly available at the Science Data Bank (42).

### Immunofluorescence confocal microscopy

PK-15 cells were seeded into confocal dishes and infected with TGEV (MOI = 0.01) or mock-infected. At the indicated time, cells were fixed with 4% paraformaldehyde for 15 min, permeabilized with 0.5% Triton X-100, and blocked with 5% skim milk for 1 h at room temperature. The cells were further incubated with primary antibodies (Abcam, UK), followed by appropriate secondary antibodies (Abbkine, China). Nuclei were stained with DAPI (Beyotime, China). Images were acquired using a Leica TCS SP8 confocal microscope.

### Quantification of m^6^A Levels

Global m^6^A levels were quantified using the EpiQuick m^6^A RNA Methylation Quantification Kit (EpiGentek, USA) according to the manufacturer’s instructions. Briefly, the RNA extracted from purified virions or cells was bound to assay wells. After washing with the wash buffer, the samples were first incubated with the supplied capture antibodies and further with the detection antibodies. Finally, the signal was measured at 450 nm. Triplicate samples were used for each condition, and m^6^A percentages were calculated using the supplied formula.

### Dot blot analysis

Dot blot assays were conducted as previously described (43). Total RNA (make up to 100 μL using 1 mM EDTA) was mixed with 60 μL of 20× saline-sodium citrate buffer (3 M NaCl, 0.3 M trisodium citrate) and 40 μL of 37% formaldehyde, incubated at 65°C for 30 min. Equal amounts of RNA were spotted onto nitrocellulose (for m^6^A detection) and nylon membranes (for loading control). The nylon membrane was stained with methylene blue (Sigma, Germany), while the nitrocellulose membrane (Beyotime, China) was UV cross-linked (254 nm, 30 min) and blocked with 5% milk. M^6^A was detected using an anti-m^6^A antibody (1:5,000; Synaptic Systems, Germany) and Dylight 800-conjugated secondary antibody (1:20,000; Abbkine, China). Signals were visualized using the Odyssey CLX imaging system (LI-COR, USA).

### RNA immunoprecipitation

PK-15 cells were transfected with expression plasmids or an empty vector, then infected with TGEV (MOI = 0.1). At 36 hpi, cells were UV cross-linked (150 mJ/cm², 254 nm) and lysed. The lysates were incubated with anti-Myc antibody, protein G magnetic beads (Biodragon, China), and RNase inhibitor (Roche, Switzerland) at 4°C for 2 h. Beads were washed, and RNA was extracted using RNAiso Plus (TaKaRa, Japan) and analyzed by RT-qPCR.

### CCK-8 cell viability assay

Cell viability was measured using the CCK-8 kit (Dojindo, Japan) following the manufacturer’s instructions. Briefly, PK-15 cells were seeded into 96-well plates (1 × 10⁴ per well). After 12 h, cells were treated with 3-DAA at various concentrations and incubated for another 8 h. The CCK-8 reagent was then added (10 μL per well) and incubated for another 1 h. Absorbance at 450 nm was recorded at designated time points.

### RNA stability assay

Cells were transfected with siRNAs (100 nM) and/or treated with Remdesivir (6 μM; Ambeed, China) before TGEV infection (MOI = 0.01). Total RNA was extracted at 6, 12, and 18 hpi. Both GAPDH and viral RNA were quantified by RT-qPCR. The expression of TGEV N in PK-15 cells treated with control siRNA and DMSO was used as a normalization control.

### Luciferase reporter assay

PK-15 cells were seeded in 24-well plates and transfected with 250 ng of luciferase reporter plasmids for interferon (IFN)-β, NF-κB, IRF3, or IRES, along with 50 ng of Renilla luciferase plasmid (Promega, USA). The luciferase reporter plasmids are commercially available (Beyotime, China). The response element of IFN-β/NF-κB/IRF3 is placed ahead of the luciferase, while the IRES sequence is placed ahead of the luciferase promoter. After 12 h, cells were treated with 3-DAA or STM2457 and infected with TGEV (MOI = 0.01) or mock-infected. Luciferase activity was measured using the Dual-Glo Luciferase Assay System (Promega, USA) at 24 hpi.

### Real-time PCR

Viral RNA levels were quantified by RT-qPCR targeting the TGEV N gene (44) using TransStart Probe qPCR SuperMix (TransGen, China) on a Bio-Rad CFX96 system. Host gene expression was measured using a SYBR Green qPCR Master Mix (Novogen, China). All reactions were run in triplicate. Relative gene expression was calculated using the ∆∆Ct method. Primer sequences are listed in Table 2.

**TABLE 2:** The primers used for m^6^A real-time PCR analysis

Gene	Prime sequence
MDA5	F: TTCTGCCTGCAGAGGTGAAG R: AGAGCCTGCACAAACATCCT
RIG-I	F: ACTTACAGCCCACTGAAGCC R: ACAACCTTCCCCTTTCGTCC
IFN-α	F: CAGTTCTGCACTGGACTGGA R: CACAGGGGCTGTAGCTCTTC
IFN-β	F: GCTAACAAGTGCATCCTCCAAA R: CCAGGAGCTTCTGACATGCCA
IFN-γ	F: GAATTGGAAAGAGGAGAGTGACAGA R: GTCTCCACACTCTTTTGGATGCT
IFN-λ1	F: TGTCACCACAGGAGCTGAAG R: TAGCTCAGCCTCTAAGGCCA
IFN-λ3	F: TTGGCCCAGTTCAAGTCTCT R: GAGCTGCAGTTCCAGTCCTC
GAPDH	F: CAAGAAGGTGGTGAAGCAGG R: ACCAGGAAATGAGCTTGACG

### RNA pull-down assay

Approximately 20 µg of viral RNA extracted from purified virions was biotinylated (20106, Thermo, USA) and then performed with RNA pulldown kit (DLI201, Qinke, China) according to the manufacturer’s instructions. Briefly, biotinylated RNA was first incubated at 90°C for 2 min, followed by 4°C for 2 min, and finally mixed with streptavidin beads at 20°C for 30 min in the presence of RNase inhibitor. After washing, beads were divided into three parts and incubated with lysates from 293T cells overexpressing Myc-tagged RIG-I, MDA5, or empty vector for 1 h at 20°C. Bound proteins were eluted at 65°C for 10 min. The supernatant was finally analyzed by western blotting using anti-Myc antibody (Biodragon, China).

### Generation of RIG-I knockout PK-15 cells

CRISPR/Cas9-mediated knockout of porcine RIG-I was performed using sgRNA targeting exon 1. The sgRNA was designed via an online CRISPR tool (http://crispr.mit.edu/) and cloned into pX459 vector. After transfection, cells were selected by puromycin (2 μg/mL), cloned by limiting dilution, and screened by PCR and Sanger sequencing. RIG-I protein expression was validated by western blotting using specific antibodies (Cell Signaling Technology, USA).

### Virus purification

Culture supernatants containing TGEV were clarified by centrifugation at 2,000 × *g* for 10 min at 4°C. The supernatant was ultracentrifuged at 100,000 × *g* for 2 h. Pellets were resuspended in PBS and subjected to sucrose density gradient ultracentrifugation (20%, 40%, 50%). The virion-containing layer (between 40% and 50%) was collected, diluted in PBS, and pelleted again by ultracentrifugation at 100,000 × *g* for 2 h. The purity and intensity of vRNAs were further tested by qPCR and presented in sFig. 2.

### Statistical analysis

All quantitative data were analyzed using Student’s *t*-test. *P* <0.05 was considered statistically significant, and *P* <0.01 was considered highly significant. “NS” indicates no significant difference.

## RESULTS

### The TGEV genome is modified by m^6^A methylation

**Fig 1:**
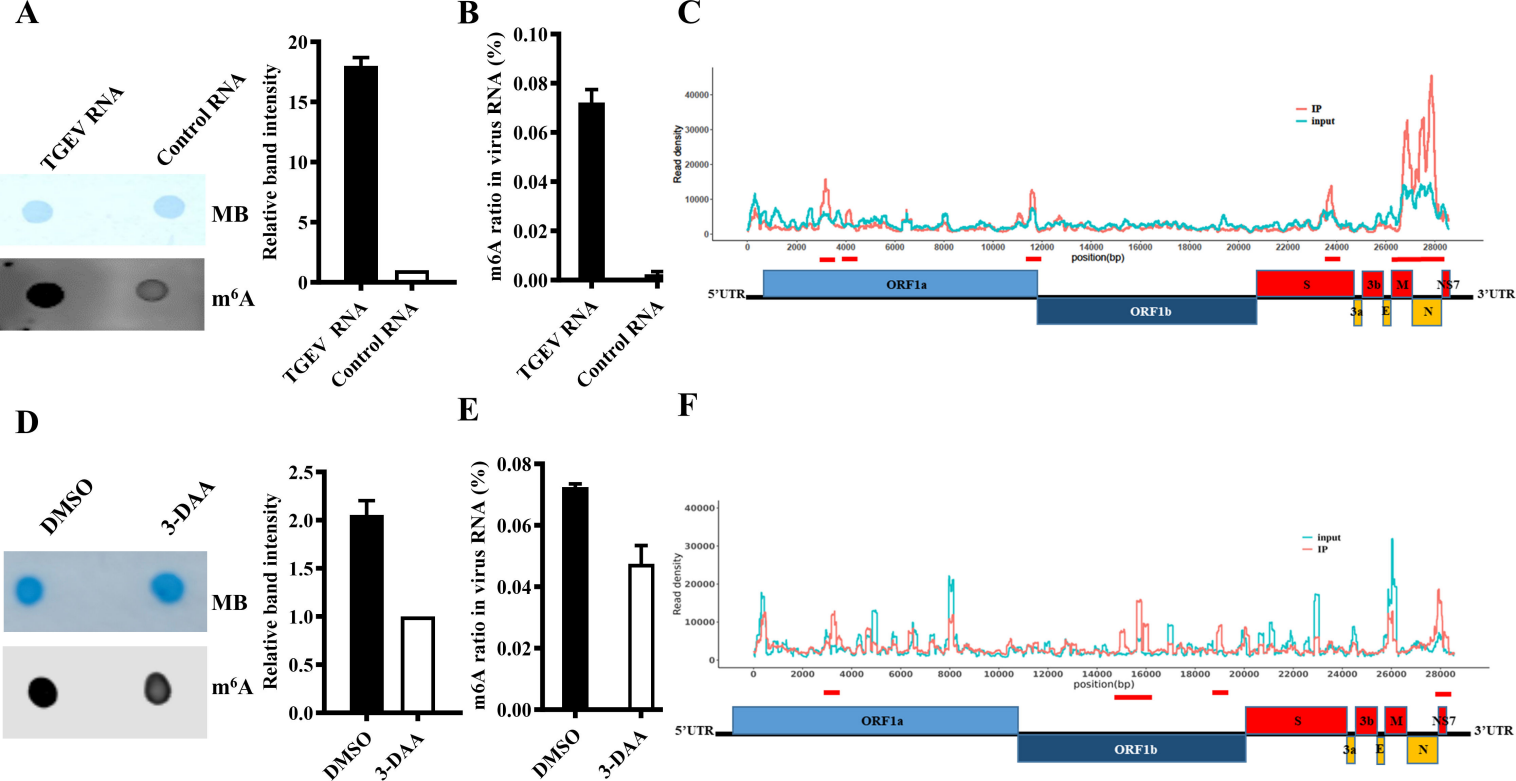
M^6^A modification is present in the TGEV genome. (**A**) A total of 600 ng TGEV genomic RNA was extracted from purified virions and subjected to m^6^A quantification by dot blot. (**B**) ELISA quantification of m^6^A levels using the same RNA. Synthetic scrambled RNA served as a negative control. (**C**) M^6^A-seq was performed on RNA extracted from TGEV-infected or mock-infected cells to map m^6^A sites. Red and blue lines represent m^6^A-IP and input RNA-seq reads, respectively. Red bars on the TGEV genome indicate significant m^6^A peaks identified in two independent experiments using MeRIPPeR (*P* < 0.05). (**D–E**) Both dot blot and ELISA revealed a reduction in the m^6^A ratio of TGEV RNA (600 ng) extracted from purified virions upon treatment with 3-DAA. (**F**) M^6^A peak distribution and enrichment patterns were also markedly altered in 3-DAA-treated cells.

To determine whether the TGEV genome is modified by m^6^A, viral RNA was extracted from purified TGEV-H virions. As a negative control, RNA of comparable quality was obtained from *in vitro* transcription (IVT). Both RNA samples were first stained with methylene blue as a loading control, followed by m^6^A dot blot analysis. The virion RNA exhibited significantly higher m^6^A levels compared to IVT RNA, indicating the presence of m^6^A modifications in the TGEV genome (Fig. 1A). Quantification using an m^6^A RNA methylation assay kit revealed that the m^6^A ratio in TGEV RNA ranged from approximately 0.065% to 0.078% (Fig. 1B). Immunoprecipitation with anti-m^6^A antibodies followed by sequencing identified six prominent m^6^A peaks distributed across ORF1a, S, M, N, and NSP7 regions (Fig. 1C; Table 3).

**TABLE 3:** The peaks identified in TGEV

Peak position	*P* value	Enrichment	GENE
3,995–4,245	0.0028	2.131994128	ORF1a
23,561–23,912	0.00018	2.382262025	S
23,211–23,412	0.00018	2.173551708	S
3,099–3,548	0.00016	2.414750592	ORF1a
26,762–26,862	2.80E-06	2.756547173	M
27,924–28,323	2.60E-07	2.913273207	N, NSP7

To assess the functional role of methylation, PK-15 cells were treated with the methylation inhibitor 3-DAA prior to TGEV infection. Dot blot and ELISA assays showed that the m^6^A ratio in TGEV genome RNA decreased from ~0.07% to ~0.05% after 3-DAA treatment (Fig. 1D and E). Furthermore, m^6^A-seq analysis demonstrated altered distribution and reduced enrichment of m^6^A-modified regions, particularly within the M, N, and NSP7 genes (Fig. 1F). The enrichment scores for M and N/NSP7 regions declined from 2.75 and 2.91, respectively, to 0.97 and 1.98 after treatment (Table 4). These results confirm that TGEV genomic RNA is modified by m^6^A, and this modification is dynamic and sensitive to cellular methylation levels.

**TABLE 4:** The peaks identified in TGEV treated with 3-DAA

Peak position	*P* value	Enrichment	GENE
3,962–4,133	0.00018	2.231556326	ORF1a
15,432–15,732	0.00018	2.122234267	ORF1b
15,893–16,121	0.00018	2.053431135	ORF1b
18,573–18,762	0.00018	1.973436782	ORF1b
28,124–2,8322	0.00018	1.983456273	N, NSP7

### M^6^A methylation suppresses TGEV replication

**Fig 2:**
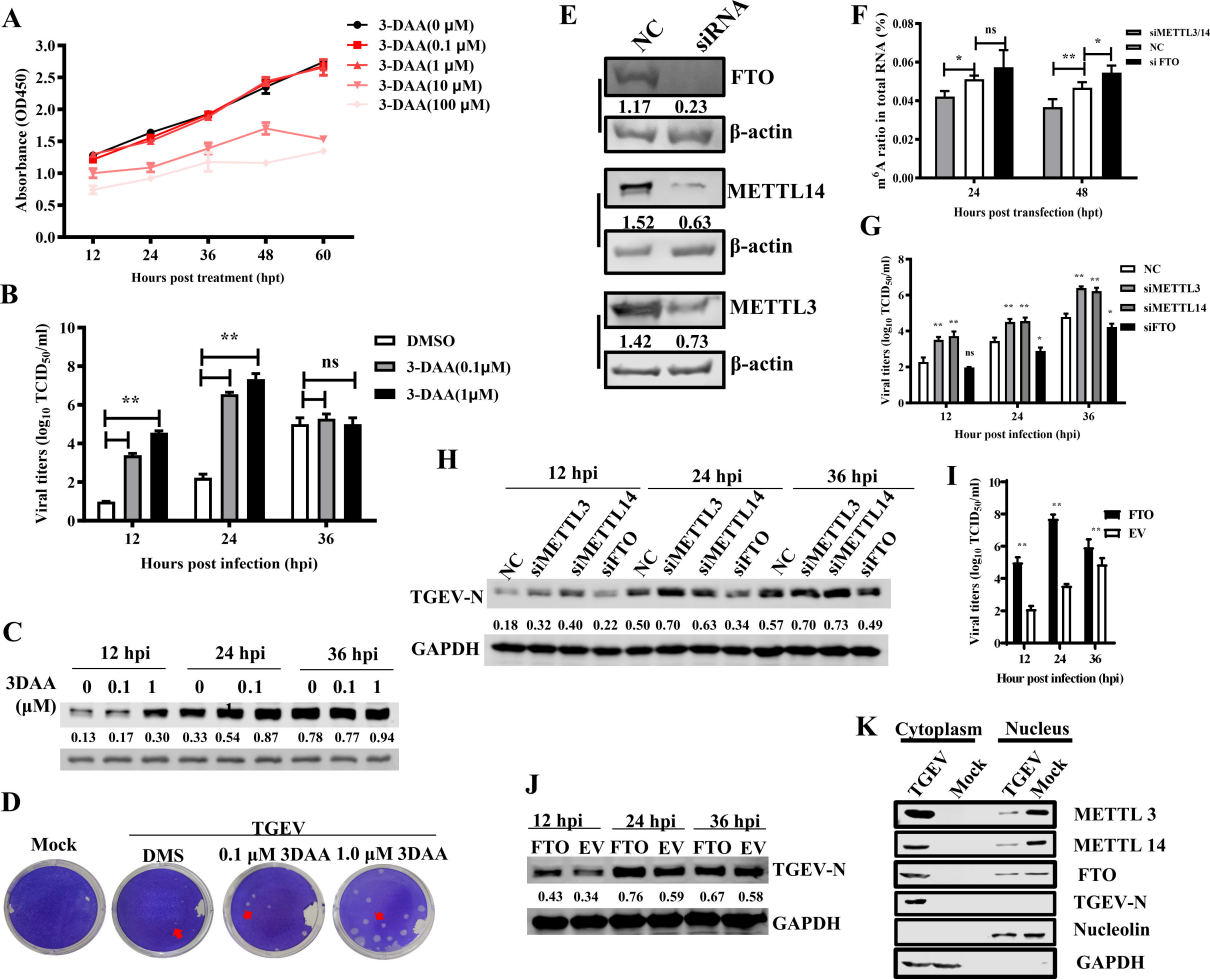
M^6^A modification suppresses TGEV replication. Cell viability of PK-15 cells treated with various concentrations of 3-DAA was assessed using a CCK-8 assay. (**B**) TGEV growth kinetics in PK-15 cells treated with different concentrations of 3-DAA. (**C**) TGEV N protein expression at different time points was detected by western blotting analysis, with relative intensity indicated. (**D**) Plaque assay of 3-DAA-treated cells infected with TGEV. (**E**) The knock-down efficiency test of siRNAs against METTLs or FTO, with relative intensity indicated. PK-15 cells transfected with siRNAs (100 nM) were harvested at 48 hpt and detected by western blotting analysis. (**F**) m^6^A ratio of the total RNA in siRNA-transfected cells tested by ELISA quantification. (**G**) Viral titers in culture supernatants derived from PK-15 cells transfected with siRNAs (100 nM). (**H**) Western blot analysis of TGEV N protein in PK-15 cells transfected with siRNAs (100 nM) at 12, 24, and 36 hpi, with relative intensity indicated. (**I–J**) PK-15 cells transfected with FTO-overexpressing plasmids were infected with TGEV for viral titer measurement (**I**) and N protein detection (**J**) at 12, 24, and 36 hpi. (**K**) Subcellular localization of FTO, METTL3, and METTL14 in nuclear and cytoplasmic fractions of PK-15 cells. *P < 0.05, **P < 0.01.

To explore the functional consequences of m^6^A modification on viral replication, the cytotoxicity of 3-DAA was first evaluated in PK-15 cells using the CCK-8 assay. Cell viability was significantly reduced at concentrations between 10 and 100 µM (Fig. 2A). Therefore, lower concentrations (0.1 and 1 µM) were used to treat cells infected with TGEV at an MOI of 0.01. Growth kinetics revealed that 3-DAA treatment significantly enhanced viral replication by over 100-fold at 12 and 24 hpi compared to control (Fig. 2B). Western blotting of TGEV N protein confirmed these findings (Fig. 2C), as did plaque assays, which showed an increase in both plaque size and number following 3-DAA treatment (Fig. 2D).

To further assess the role of methylation machinery, siRNAs targeting porcine METTL3, METTL14 (methyltransferases), and FTO (a demethylase) were transfected into cells, followed by TGEV infection. Western blotting confirmed successful knockdown (Fig. 2E). Knockdown of METTL3/14 reduced while knockdown of FTO increased the m^6^A level (Fig. 2F). Consistently, TGEV replication was enhanced upon METTL3/14 knockdown and suppressed by FTO knockdown, as determined by viral titration and western blotting of TGEV N (Fig. 2G and H). Overexpression of Myc-tagged FTO further increased viral titers and N protein expression (Fig. 2I and J).

**Fig 3:**
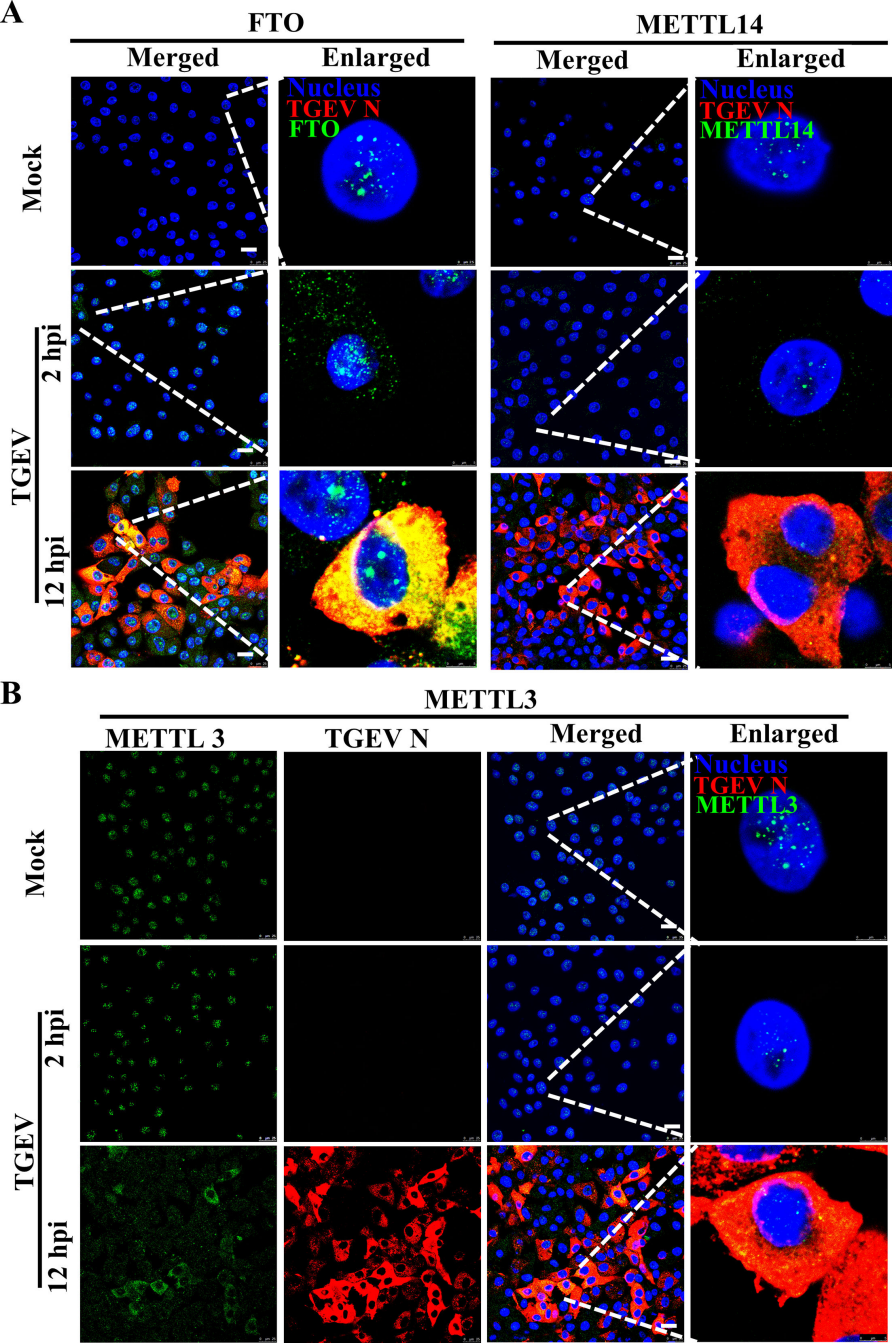
TGEV infection changes the subcellular localization of FTO, METTL3, and METTL14. PK-15 cells were infected or mock-infected with TGEV (MOI = 0.01) and prepared for confocal analysis. The cells were harvested at 2 and 12 hpi, stained for FTO or METTL14 (**A**) and METTL3 (**B**). Scale bars: 25 μm.

Moreover, the subcellular fraction showed that the translocation of METTLs and FTO changed during TGEV infection (Fig. 2K). METTL3, METTL14, and FTO were localized in the nucleus under basal conditions but translocated to the cytoplasm upon TGEV infection, indicating that m^6^A methylation and demethylation of TGEV RNA occur primarily in the cytoplasm. Immunofluorescence further confirmed the translocation. Confocal analysis showed that these proteins aggregate in the nucleus to form dense granules in all cells before infection, but are scattered in the cytoplasm after infection. The intensity of FTO was also significantly elevated, suggesting TGEV infection induced its up-regulation. However, METTL3 was observed in all cells at 2 hpi and the control cells, but only presented in much fewer cells at 12 hpi, suggesting it was degraded since TGEV infection (Fig. 3A and B).

### M^6^A reader proteins negatively regulate TGEV replication

**Fig 4:**
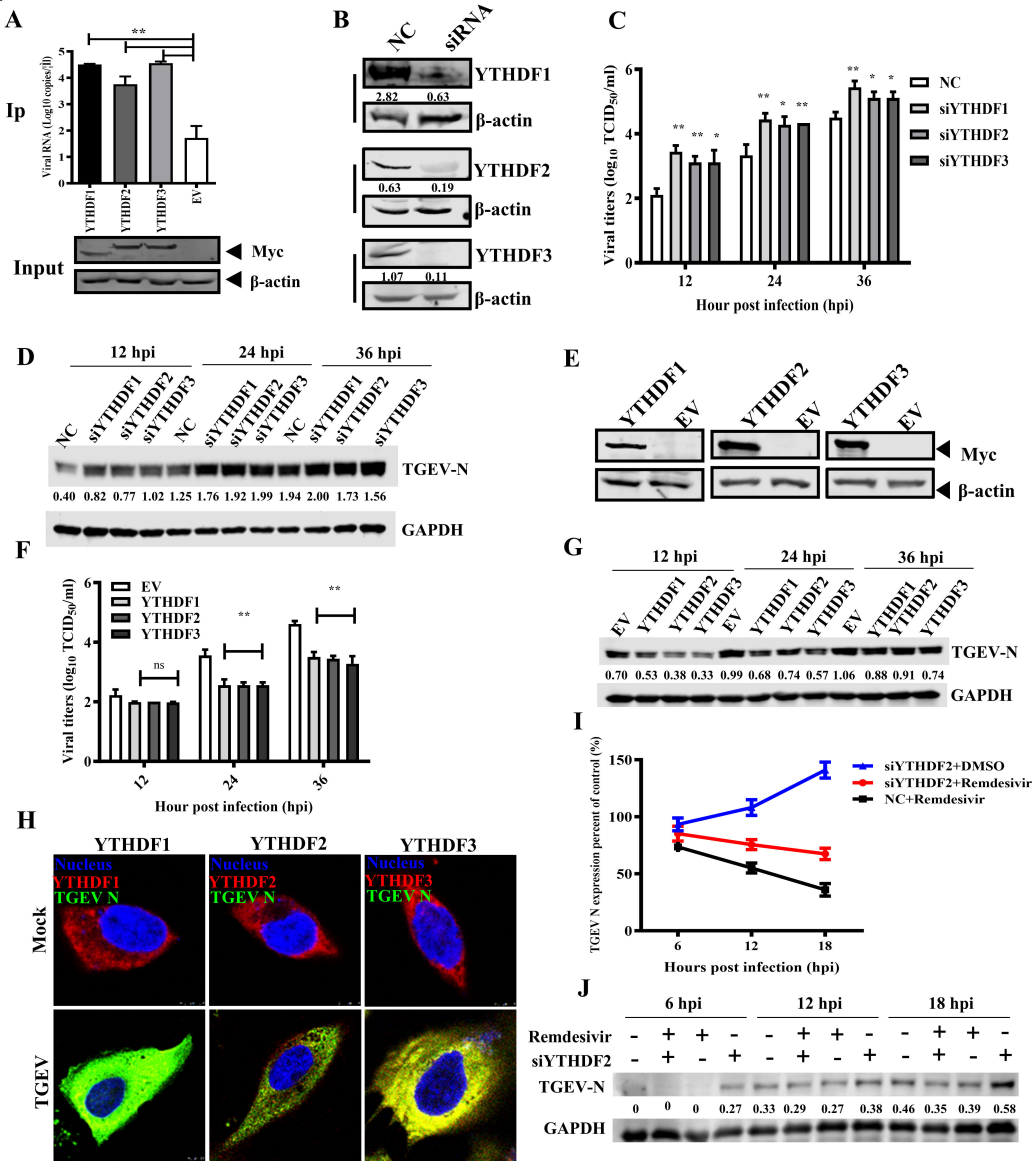
YTHDF proteins negatively regulate TGEV replication. (**A**) The RNA affinity testing between YTHDFs and TGEV genomic RNA. PK-15 cells were transfected with Myc-YTHDF1–3 and then infected with TGEV of MOI 0.01. The cells were then UV cross-linked at 36 hpi and subjected to RNA immunoprecipitation analysis. The affinity of YTHDFs to TGEV genomic RNA was assessed by RT-qPCR. (**B**) The siRNA efficiency against YTHDFs was validated by western blotting, with relative intensity indicated. (**C**) The viral growth kinetics of PK-15 cells transfected with siRNAs (100 nM) targeting YTHDFs or control siRNA. (**D**) TGEV N protein levels in siRNA-treated cells, with relative intensity indicated. (**E–G**) Western blot, growth kinetics, and N protein levels in PK-15 cells transfected with YTHDF-expressing plasmids or empty vector (EV). (**H**) Confocal microscopy of YTHDF1–3 in TGEV- or mock-infected cells at 24 hpi. (**I**) The RNA decay curve of TGEV genome RNA subjected to YTHDF2 knockdown and Remdesivir treatment. The cells infected with TGEV, treated with control siRNA and DMSO, were taken as a control. (**J**) Western blotting analysis of TGEV N protein in PK-15 cells treated with siRNA against YTHDF2 or Remdesivir, with relative intensity indicated. *P < 0.05, **P < 0.01.

To evaluate the role of m^6^A reader proteins, Myc-tagged porcine YTHDF1, YTHDF2, and YTHDF3 were overexpressed in PK-15 cells, followed by TGEV infection. RNA immunoprecipitation (RIP) and qPCR showed strong binding (over 100-fold enrichment) of all three YTHDF proteins to TGEV RNA compared to vector controls (Fig. 4A). siRNA-mediated knockdown of YTHDFs was validated by western blotting (Fig. 4B), and knockdown resulted in enhanced viral replication, as shown by growth curves and increased N protein levels (Fig. 4C and D). Conversely, overexpression of YTHDFs suppressed viral replication (Fig. 4E through G). Confocal microscopy showed that YTHDF1–3 localized primarily in the cytoplasm and did not change location upon TGEV infection (Fig. 4H through J), supporting a cytoplasmic role for m^6^A-mediated viral RNA regulation.

YTHDF2 is known to reduce the stability of m^6^A-modified transcripts. To evaluate its effect on TGEV RNA stability, cells were transfected with siRNAs against YTHDF2 and treated with DMSO or the viral polymerase inhibitor Remdesivir (6 µM) (45). The copies of the TGEV genome in PK-15 cells treated with control siRNA and DMSO were used as the normalization control for RNA decay curve depiction. The results revealed that Remdesivir (Remdesivir+NC) significantly reduced viral RNA, whereas YTHDF2 knockdown (Remdesivir+siYTHDF2) led to elevated RNA levels, indicating that YTHDF2 suppresses TGEV by destabilizing its RNA (Fig. 4I). Western blotting of TGEV N protein further confirmed the results above (Fig. 4J).

### TGEV infection alters host m^6^A RNA methylation patterns

**Fig 5:**
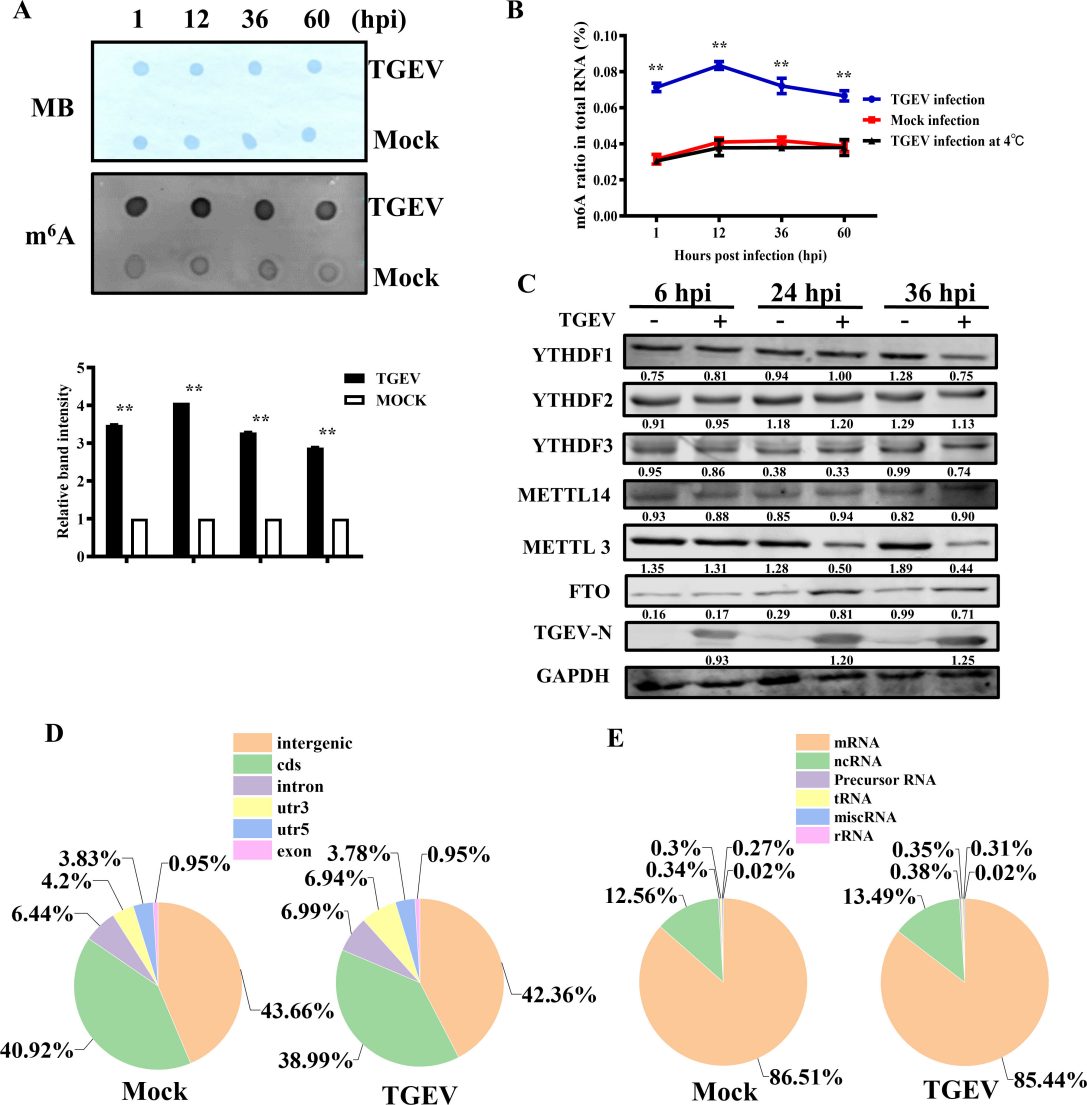
TGEV infection enhances m^6^A modification by modulating METTLs and FTO expression. (**A**) Dot blot analysis of total RNA extracted from TGEV-infected and mock-infected cells at various time points. Relative m^6^A levels are shown as band intensity. (**B**) ELISA-based quantification of m^6^A levels in total RNA extracted from cells at various time points following TGEV infection at 4°C. (**C**) Western blot analysis of m^6^A methyltransferases, demethylases, and readers in infected vs mock-infected cells, with relative intensity indicated. Distribution of m^6^A peaks across host mRNA regions (**D**) and RNA transcript types (**E**) changed during TGEV infection. *P < 0.05, **P < 0.01.

Since m^6^A is also present in host RNAs, we investigated whether TGEV infection affects host methylation dynamics. Dot blot analysis of the host RNA demonstrated a marked increase in total m^6^A levels following TGEV infection (Fig. 5A). Quantification showed m^6^A peaked at 24 hpi before slightly declining (Fig. 5B). In contrast, cells infected at 4°C did not show increased m^6^A, suggesting viral replication is required for the boost of m^6^A level. Western blotting revealed upregulation of FTO and downregulation of METTL3 post-infection, while METTL14 and YTHDF1–3 expression remained largely unchanged, which correlated with the results of Fig. 3 (Fig. 5C). M^6^A-seq also showed that TGEV infection changed the m^6^A topology by decreasing its location among mRNA and increasing its location among 3-UTR, which is the main functional region for m^6^A to control gene expression (Fig. 5D and E). These modifications highlight the crucial role of m6A methylation, indicating that TGEV infection modulates host m6A methylation patterns and the expression of METTL3 and FTO.

### M^6^A modification activates the interferon signaling pathway

**Fig 6:**
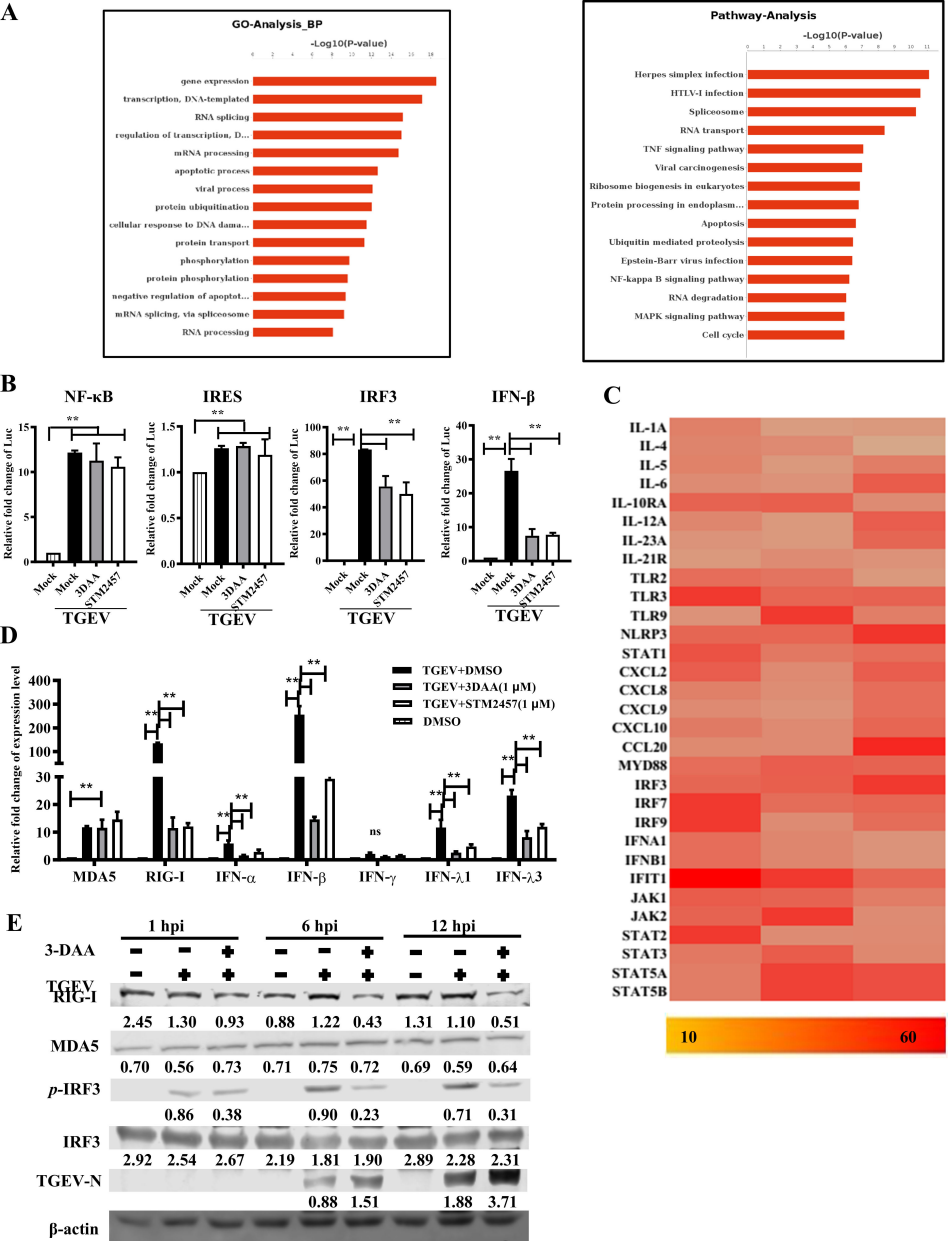
Enhanced m^6^A modification activates the interferon signaling pathway. (**A**) GO and KEGG pathway enrichment analyses of m^6^A-modified mRNAs. (**B**) Luciferase reporter assays in PK-15 cells infected with or without TGEV and treated with 3-DAA or STM2457. (**C**) Heatmap showing m^6^A-modified mRNAs highly involved in interferon and cytokine production. All genes presented have a fold change >10 and are supported by ≥3 sequence fragments. (**D**) RT-qPCR analysis of interferons and RNA sensors in cells infected with or without TGEV and treated with 3-DAA or STM2457. Gene expression was normalized to GAPDH and calculated using the 2^−ΔΔCt^ method. (**E**) Western blotting analysis of TGEV-infected or mock-infected cells treated with 3-DAA, with relative intensity indicated. “**” indicates highly significant. “ns” indicates no significant difference.

To identify the functional consequences of host m^6^A methylation changes, Gene Ontology (GO) and KEGG pathway analyses were conducted on m^6^A-seq data. The results showed the enriched categories included RNA metabolism and viral infection pathways (Fig. 6A). Dual-luciferase assays of NF-κB, IRES, IRF3, and IFN-β reporter constructs showed significant upregulation upon TGEV infection. Notably, 3-DAA and STM2457 (a METTL3 inhibitor) showed no inhibitory effect on IRF3 and IFN-β activity without TGEV infection (data not shown), but a strong reduction after TGEV infection, suggesting m^6^A regulates IFN pathway activation (Fig. 6B). The genes highly enriched by MeRIP with over 10-fold change after normalization to input were subsequently analyzed, which included IFN-α, IFN-β, and IRF3/7/9 (Fig. 6C). qPCR and western blotting confirmed these findings, showing downregulation of IFN-λ1, IFN-λ3, RIG-I, and p-IRF3 upon 3-DAA or STM2457 treatment (Fig. 6D and E). These results indicate m^6^A methylation enhances IFN signaling during TGEV infection.

### RIG-I senses m^6^A-modified TGEV RNA

**Fig 7:**
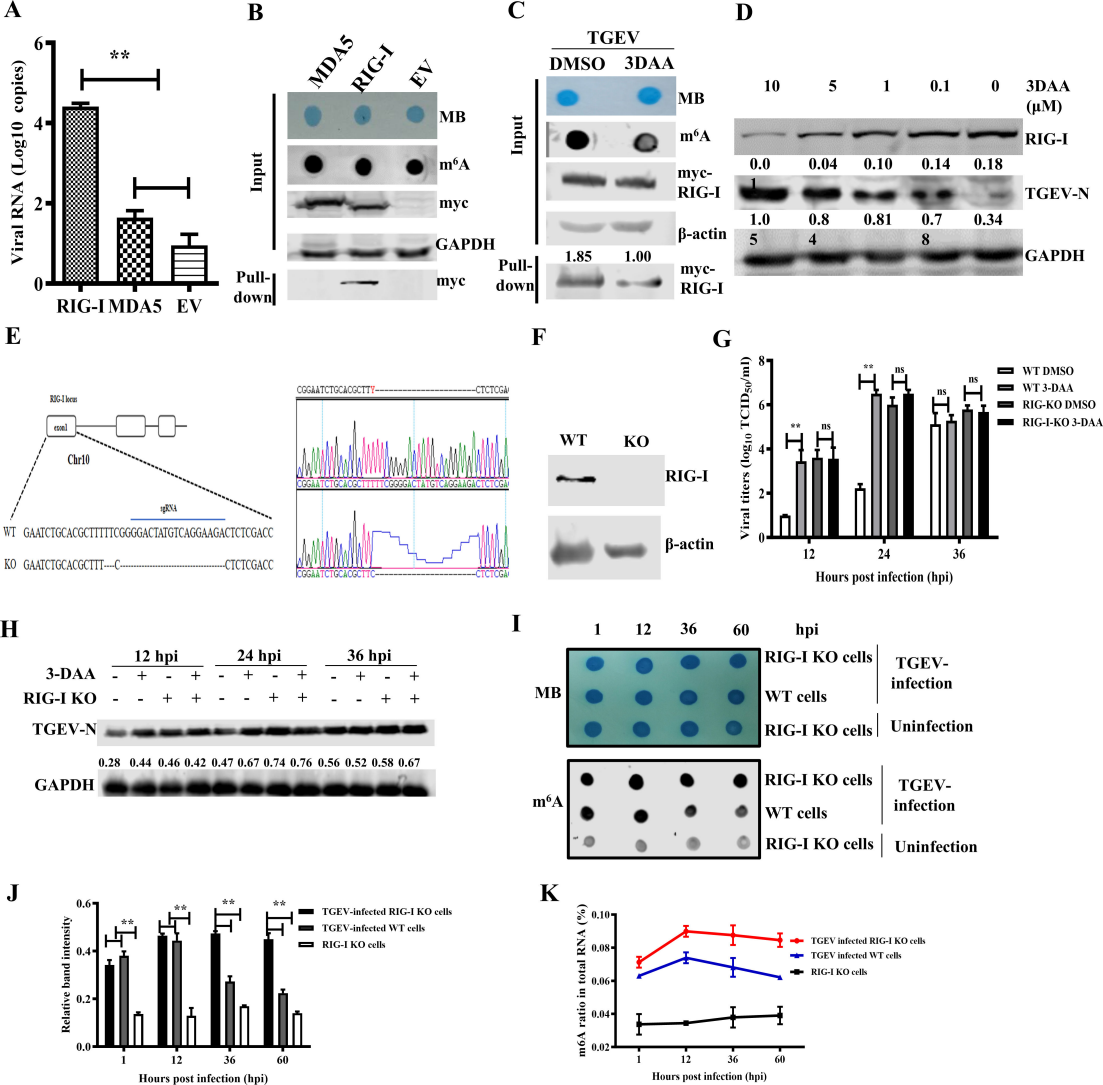
Recognition of TGEV RNA by RIG-I depends on m^6^A modification. (**A**) The RNA affinity testing between RNA sensors and TGEV genomic RNA by RNA immunoprecipitation analysis. PK-15 cells were transfected with Myc-RIG-I or Myc-MDA5 and then infected with TGEV of MOI 0.01. The cells were then UV cross-linked at 36 hpi and subjected to RNA immunoprecipitation analysis. The affinity of RIG-I/MDA5 to TGEV genomic RNA was assessed by RT-qPCR. (**B**) RNA pull-down analysis shows RNA sensors displayed with different affinities with TGEV RNA. The vRNA was extracted from purified virions and biotinylated, which was further incubated with cell lysate to capture the targets and detected by western blotting. (**C**) RNA pull-down analysis using TGEV RNA extracted from virions, which were harvested from cells treated with 3-DAA or DMSO. (**D**) 3-DAA treatment facilitates TGEV growth and inhibits RIG-I expression. (**E–F**) Sequencing and western blotting validation of RIG-I knockout (KO) and wild-type (WT) PK-15 cells. (**G–H**) TGEV growth kinetics and N protein expression in KO and WT cells treated with 3-DAA or DMSO. (**I–J**) Dot blot and its density analysis of m^6^A levels in total RNA extracted from KO and WT cells infected with or without TGEV. (**K**) ELISA analysis of m^6^A levels in total RNA extracted from KO and WT cells infected with or without TGEV. “**” indicates highly significant. “ns” indicates no significant difference.

To determine the RNA sensor responsible for recognizing m^6^A-modified TGEV RNA, Myc-tagged RIG-I and MDA5 were overexpressed in 293T cells. At 36 hpi, the cells were UV-cross-linked and lysed. The supernatant was first incubated with anti-Myc antibodies, protein G beads, and RNase inhibitor at 4°C for 2 h and then washed with lysis buffer. The vRNA binding to RNA sensors was extracted and quantified via qPCR. The result revealed more TGEV RNA bound to RIG-I (Fig. 7A) while nearly the same number of TGEV RNA was taken as a control. We further used RNA pull-down to detect RNA sensors binding to viral RNA. All viral RNA was extracted from purified virions, which were of the same intensity and without host RNA contamination (data not shown). The result confirmed this preference that vRNA can pull down RIG-I (Fig. 7B). When qualified TGEV RNA was harvested from 3-DAA-treated cells (with reduced m^6^A), RIG-I binding was attenuated (Fig. 7C). Increasing 3-DAA concentrations were also found to promote TGEV replication and decrease the expression of RIG-I (Fig. 7D).

To further confirm the role of RIG-I, a RIG-I knockout (KO) PK-15 cell line was generated via CRISPR/Cas9 and verified by sequencing and western blotting (Fig. 7E and F). Viral replication was significantly affected by 3-DAA in wild-type (WT) cells but not in KO cells (Fig. 7G and H), confirming RIG-I mediates m^6^A-dependent sensing of TGEV. 3-DAA treatment posed little influence on TGEV growth in RIG-I KO cells, further suggesting the sensing and following response were m^6^A-dependent (Fig. 7G and H). Dot blot and m^6^A quantification showed that m^6^A levels of total host RNA declined after 36 hpi in WT but remained stable in KO cells (Fig. 7I through K), suggesting RIG-I also contributes to feedback regulation of RNA methylation.

### Abnormally high m^6^A levels in TGEV RNA facilitate its sensing by RIG-I

**Fig 8:**
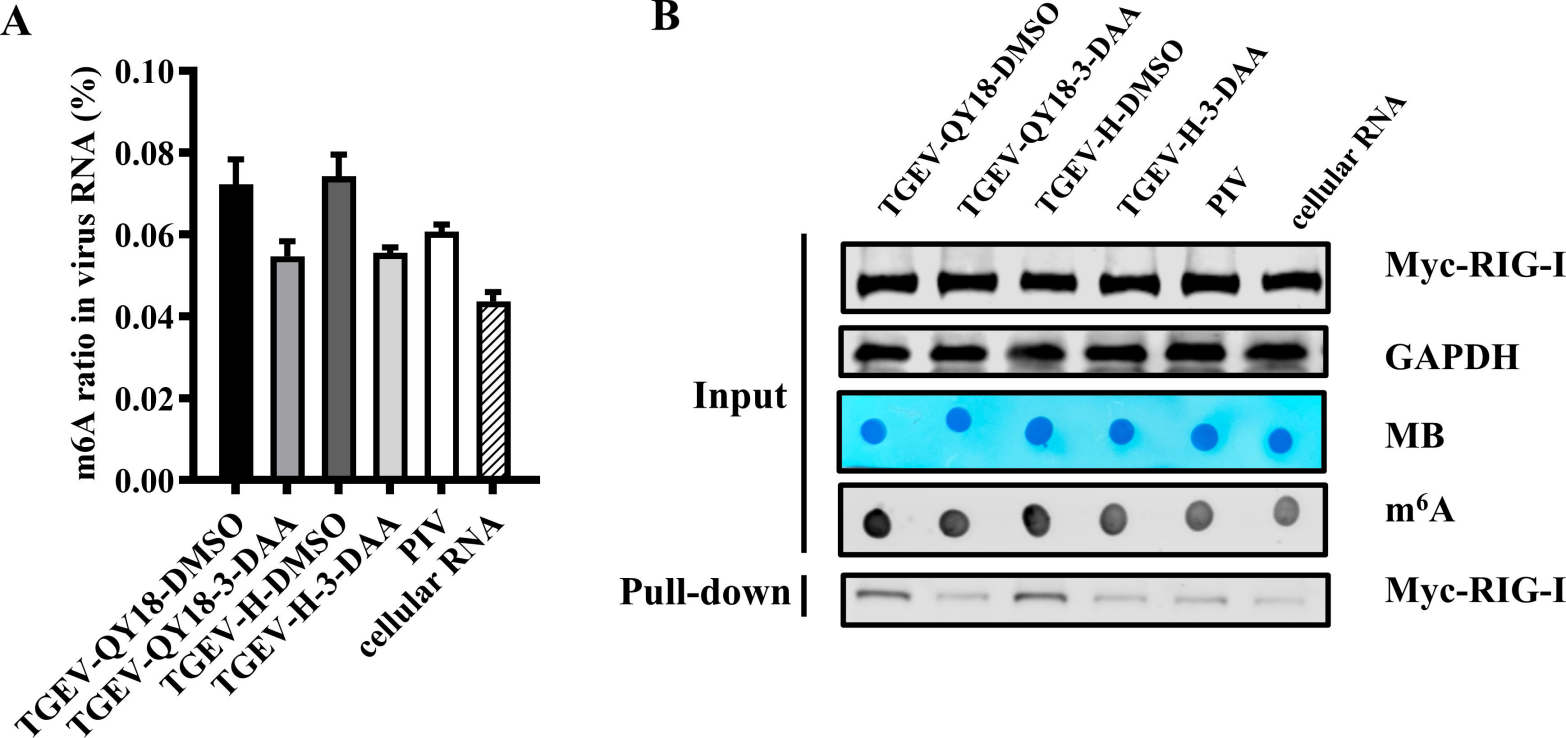
Increased m^6^A modification of TGEV RNA enhances RIG-I recognition. (**A**) ELISA quantification of m^6^A levels in viral RNA extracted from purified virions harvested from DMSO- or 3-DAA-treated cells, and host RNA. (**B**) RNA pull-down assays reveal differential RIG-I binding affinity to various viral RNAs. All viral RNAs were extracted from purified virions.

To assess how m^6^A density affects recognition by RIG-I, RNAs from TGEV-H, TGEV-QY18, parainfluenza virus (PIV), and host RNA were analyzed. TGEV RNA from DMSO-treated cells had the highest m^6^A levels (~0.07%), decreasing to ~0.05% after 3-DAA treatment. PIV RNA from virions had ~0.06% m^6^A, while host RNA had ~0.04% (Fig. 8A). Pull-down assays revealed that TGEV RNA with higher m^6^A had greater affinity to RIG-I, whereas viral RNA with m^6^A level close to host RNA showed much fainter RIG-I binding, consistent with previous studies (Fig. 8B) (24, 25). These findings indicate that the abnormally high m^6^A density in TGEV RNA enhances RIG-I recognition, contributing to antiviral sensing and suppression of viral replication.

## DISCUSSION

**Fig 9:**
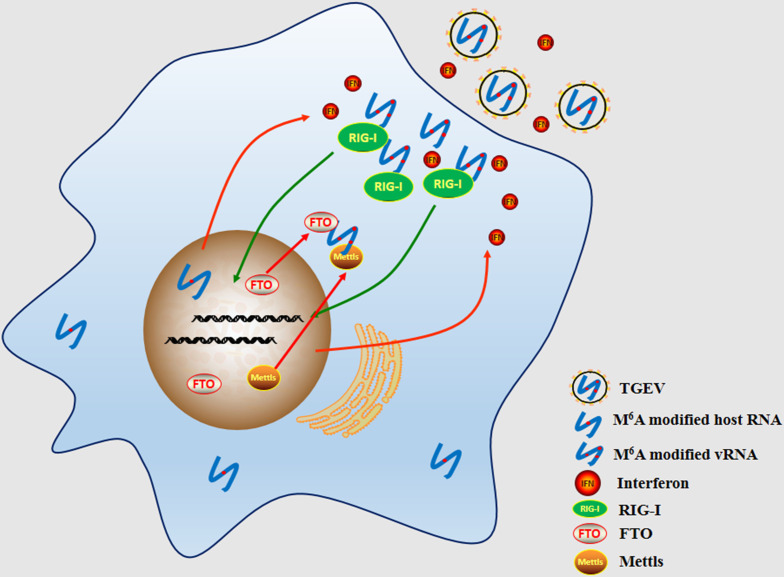
Schematic model of m^6^A-mediated antiviral response. Schematic representation of how m^6^A modification enhances the recognition of TGEV RNA by RIG-I, leading to interferon production and inhibition of viral replication.

M^6^A is one of the most prevalent epigenetic modifications on RNA, playing essential roles in gene regulation. In this study, we demonstrate that m^6^A modification inhibits the replication of TGEV. Mechanistically, the m^6^A reader protein YTHDF2 binds to m^6^A-modified sites on TGEV RNA, promoting its degradation and reducing viral RNA stability. More importantly, we show that the unusually high m^6^A ratio of TGEV RNA compared to other viruses can facilitate the detection of the pattern recognition receptor RIG-I, thereby activating the innate immune response and inducing IFN and pro-inflammatory cytokine production (Fig. 9). Together, our findings reveal a dual role of m^6^A in antiviral defense: destabilization of viral RNA and activation of host immunity.

To date, m^6^A modifications have been identified in over 20 viruses across nine families, yet their effects on viral replication vary. For most viruses, m^6^A enhances replication by stabilizing viral RNAs, facilitating nuclear export, or preventing immune recognition (46). However, exceptions are found among members of the *Flaviviridae* and *Coronaviridae* families, where m^6^A suppresses viral replication (32–34). This raises an important question: how does m^6^A exert opposing effects on different viruses, especially when it has been shown to help some viruses evade immune detection?

Recent studies suggest that both RNA length and m^6^A density may influence immune recognition. Stress granules preferentially bind RNAs with longer sequences or higher m^6^A content, facilitating RIG-I activation (47–49). Notably, TGEV has a large genome (>28 kb), far exceeding the length of most viral or host mRNAs. In addition, our data confirm that TGEV possesses a higher m^6^A ratio than many other viruses. These features may enhance its visibility to the immune system. Moreover, m^6^A is dynamically regulated, and its effects may vary depending on timing and context. For example, mRNA vaccines utilize pseudouridine (m1Ψ) instead of m^6^A to avoid unintended immune activation (50). Therefore, the combination of genome size, m^6^A density, and cellular context likely contributes to the differential outcomes observed among viruses.

The subcellular localization of m^6^A machinery also influences m^6^A’s effects. In host cells, m^6^A methylation is catalyzed by methyltransferases (e.g., METTL3, METTL14) and removed by demethylases (e.g., FTO, ALKBH5), which are primarily nuclear. For DNA viruses, whose replication also occurs in the nucleus, this localization remains unchanged, and m^6^A generally enhances viral gene expression (27, 29). In contrast, RNA viruses replicate in the cytoplasm. While most negative-sense RNA viruses do not appear to alter the localization of m^6^A enzymes and still benefit from m^6^A (24, 25), our study shows that TGEV—a positive-sense RNA virus—induces translocation of METTL3, METTL14, and FTO from the nucleus to the cytoplasm. This may enable direct modification of cytoplasmic viral RNA. Intriguingly, both *Flaviviridae* and *Coronaviridae*, the two virus families inhibited by m^6^A, are positive-sense RNA viruses. One exception is enterovirus 71 (EV71, *Picornaviridae*), which is also a positive-sense RNA virus but is promoted by m^6^A (22). Thus, viral genome polarity, replication site, and localization of m^6^A-related proteins collectively shape the function of m^6^A in viral infections.

Viral infections are also known to influence host m^6^A levels. Upon infection, m^6^A methylation on host transcripts often increases, promoting transcription and translation of immune-related genes and enhancing host defense—though cell-type-specific exceptions exist (26, 43, 51). In coronaviruses and other viruses, m^6^A-dependent IFN responses have been frequently observed (52–55). Our data similarly demonstrate that m^6^A promotes type I and III IFN and cytokine expression during TGEV infection. Inhibition of m^6^A with 3-DAA or STM2457 reversed these effects, supporting a role for m^6^A in the regulation of innate immunity. Mechanistically, we found that RIG-I—but not MDA5—senses m^6^A-modified TGEV RNA. Importantly, higher m^6^A density correlated with stronger RIG-I binding, in contrast to viruses like human metapneumovirus (hMPV), where m^6^A impairs RIG-I recognition (24). In hMPV, the viral RNA has a similar m^6^A density to host mRNAs, potentially allowing it to escape immune detection. These results suggest that a virus-specific threshold of m^6^A may determine whether it acts as a shield or as an immunogenic signal.

Interestingly, the distribution of m^6^A peaks along viral genomes is also virus-specific. In HIV, m^6^A is enriched in the *Gag* coding region and 3′-UTR of the genomic RNA (19–21). For flaviviruses, such as HCV and ZIKV, m^6^A is primarily found in the *NS5B*, *NS3*, and 3′-UTR regions (32, 33). In influenza virus and EV71, m^6^A is enriched in structural protein genes (22, 23), while in RSV and hMPV, m^6^A is mainly found in the G gene (24, 25). Among coronaviruses, m^6^A-seq has been reported for PEDV and SARS-CoV-2. PEDV contains fewer adenines (~24.2%–24.8%) compared to SARS-CoV-2 and TGEV (~29.1%–29.5%), possibly contributing to lower m^6^A density. PEDV exhibits m^6^A enrichment mainly in *ORF1b*, whereas in TGEV, peaks are found in *M*, *N*, and *NSP7*—genes located closer to the 3′-UTR. Notably, TGEV and SARS-CoV-2 share both similar adenine content and m^6^A peak distribution in structural genes near the 3′-UTR. The specific functions of m^6^A-modified genes remain unclear and warrant further investigation.

In summary, multiple factors—including viral RNA length, m^6^A density, genome polarity, replication site, localization of m^6^A enzymes, and host cell context—collectively determine the functional outcomes of m^6^A in viral infections. Our study reveals that m^6^A modification of TGEV RNA inhibits viral replication by reducing RNA stability and promoting RIG-I-mediated immune activation. These findings provide new insights into the antiviral roles of m^6^A and lay the groundwork for further exploration of m^6^A-based antiviral strategies.

## Data Availability

The data described in the article are available in the figures or tables.
