# Correction for Chen et al., “N6-methyladenosine within transmissible gastroenteritis virus genomic RNA inhibits its replication via efficient recognition by RNA sensor RIG-I”

**DOI:** 10.1128/jvi.00380-26

**Published:** 2026-04-27

**Authors:** Jianing Chen, Shengyu Lin, Qianzi Liu, Mengling Gao, Zemei Wang, Jiao Tang, Yaru Cui, Chen Tan, Guangliang Liu

## AUTHOR CORRECTION

Volume 100, no. 2, e01373-25, 2025, https://doi.org/10.1128/jvi.01373-25. Materials and Methods, “Virus purification” paragraph: “…and presented in sFig. 2” should read “…and results are presented in Fig. S1 at https://doi.org/10.57760/sciencedb.28945.”

